# Trans-Generational Effects of Mild Heat Stress on the Life History Traits of an Aphid Parasitoid

**DOI:** 10.1371/journal.pone.0054306

**Published:** 2013-02-06

**Authors:** Ibrahim Ismaeil, Géraldine Doury, Emmanuel Desouhant, Françoise Dubois, Geneviève Prevost, Aude Couty

**Affiliations:** 1 Université de Picardie Jules Verne, EA 4698 Ecologie et Dynamique des Systèmes Anthropisés, Equipe Bio-écologie des Insectes Phytophages et Entomophages, Amiens, France; 2 Université Claude Bernard Lyon 1, UMR CNRS 5558 Biométrie et Biologie Evolutive, Villeurbanne, France; University of Miami, United States of America

## Abstract

Temperature changes are common in nature and insects are particularly exposed and sensitive to such variations which can be potential stresses, ultimately affecting life history traits and overall fitness. Braconids have been widely used to study the effects of temperature on host-parasitoid interactions and the present work focused on the solitary endoparasitoid *Aphidius ervi* Haliday (Hymenoptera: Braconidae Aphidiidae), an efficient biological control agent commercially used against aphids such as the potato aphid *Macrosiphum euphorbiae* Thomas (Sternorrhyncha: Aphididae). Contrary to previous studies using heat shocks at extreme temperatures, we evaluated the effects of mild heat stresses by transferring young parasitoid adults from the constant temperature of 20°C to either a warm (25°C) or hot (28°C) temperature, for either 1 h or 48 h. Such treatments are consistent with situations commonly experienced by parasitoids when moved from their rearing conditions to greenhouses or field conditions. The effects were evaluated both on the heat stressed *A. ervi* adults (G0) (immediate effects) and on their first generation (G1) progeny (trans-generational effects). G0 wasps’ mortality was significantly affected by the temperature in interaction with the duration of the stress. Longevity of G0 wasps surviving the heat stress was negatively affected by the temperature and females lived longer than males. Heat stress applied to *A. ervi* parents also had consequences on their G1 progeny whose developmental time, rates of mummification and percentage of parasitoid completing total development were negatively affected. Surprisingly, the egg load at emergence of the G1 female progeny was increased when their mothers had been submitted to a mild heat stress of 25°C or 28°C. These results clearly demonstrate trans-generational phenotypic plasticity, showing that adaptation to thermal stresses may be achieved via maternal effects**.** This study also sheds light on the complexity of insect responses and underlying mechanisms to fluctuating conditions in their natural environment.

## Introduction

Stress has been defined as any environmental change that acts to reduce the fitness of an organism [1], or as a physiological disturbance that can be correlated with various abnormalities [2]. These changes include exposure to adverse biotic or abiotic environmental conditions such as pathogens, toxins, desiccation, and hot or cold temperature [3]. In organisms exposed to stress, physiological costs are likely to occur and subsequently decrease their fitness, and ultimately the fitness of the offspring. The maternal environment can be an indicator of the environmental conditions that the progeny will encounter. In such cases, maternal effects may evolve as mechanisms for trans-generational phenotypic plasticity, whereby, in response to a predictive environment, a mother may adjust the phenotypes of its progeny to the environment [4], [5].

In insects, temperature is one major abiotic factor affecting distribution, colonization, survival, abundance, behaviour and the overall fitness [6], [7]. Environmental temperatures fluctuate temporarily and spatially so insects can be exposed to many thermal stresses not only during the day but also throughout their life-cycle [8], [9]. They are particularly vulnerable to temperature variations because of their small size and ectothermic physiology [10], [7]. Most poikilothermic species are adapted to particular temperature ranges, and many studies showed that extreme temperatures, both hot and cold, can influence several of their life history traits such as mortality, development rate, adult survival, longevity, reproductive success and fecundity [1], [11]–[19]. In most studies so far reported, extreme temperatures, whether hot [20]–[22] or cold [23] were applied during a relatively short duration (except for chill-comas), corresponding to heat or cold shocks. However, under natural conditions, insects more frequently encounter less dramatic conditions. Indeed, they are repeatedly subjected to temperature variations but within narrower ranges, hence representing modest stresses rather than shocks.

Susceptibility to environmental stressors like temperature is likely to increase along with the trophic levels. This is particularly true in the case of long-term interactions such as the ones a parasitoid establishes with its host. For instance, the host/parasitoid synchrony together with the outcome of the host/parasitoid interaction can be disrupted, notably because host resistance and/or parasitoid virulence may vary depending on temperature conditions [23], [24]. The ability of natural enemies to regulate their host populations largely depends on their tolerance of environmental changes [25]. This is especially true for koinobiont parasitoids whose hosts remain alive throughout their development so that host-induced phenotypic plasticity can affect numerous wasp fitness components such as preimaginal survival, size and fecundity [26]. Braconids are widely used as models to study the effects of temperature on host-parasitoid interactions, *e.g.* [24], [27]–[31], and as they are very sensitive to stressors, species of the Aphidiinae subfamily are indicator organisms that have notably been selected for the evaluation of chemical product toxicity on non-target arthropods [32]. This study focuses on the solitary endoparasitoid *Aphidius ervi* (Haliday) (Hymenoptera: Braconidae Aphidiidae) [33]. This koinobiont parasitoid is an efficient biological control agent against cereal and vegetable aphids [34] such as the potato aphid *Macrosiphum euphorbiae* Thomas (Sternorrhyncha: Aphididae). *Aphidius ervi* is also commercially used to control several aphid species in greenhouses, where temperature conditions can be subjected to daily variations.

In most previously reported works, insects were exposed to an increasing range of constant temperatures during their development and their fitness parameters were evaluated. This allows estimation of the optimal temperature of development as well as the thermal tolerance of a species. Only a few studies report the effects of heat stress and most of them concern very hot temperatures (at least 35°C) commonly used to induce a heat shock *e.g.* [20]–[22].

Contrary to previous studies, here we evaluated the effects of mild heat stresses on young adults of *A. ervi* (G0) and on the fitness of their first generation (G1) progeny, in comparison to the constant reference temperature of 20°C. This reference temperature was chosen as *A. ervi* is commonly reared and studied at 20°C *e.g.* [35]–[37]. Several studies *e.g.* [38], [39] also show that *A. ervi* exhibits better fitness parameters between 15°C and 21°C. In our experimental conditions, mild heat stresses consisted in transferring young *A. ervi* adults from this reference temperature of 20°C to either a warm (25°C) or hot (28°C) temperature, for either a short (1 h) or longer (48 h) period of time. Surprisingly, aphid parasitoids exhibited different kinds of trans-generational phenotypic plasticity under such mild stress conditions. The implication of these findings is discussed in relation to the efficiency of parasitoid insects as biological control agents.

## Materials and Methods

### Insects

The colony of *Macrosiphum euphorbiae* (Thomas) (Homoptera: Aphididae) has been reared on *Solanum tuberosum* potato plants (cv. Désirée) in cages (35 cm×55 cm×80 cm) covered with nylon mesh since 2004. The *M. euphorbiae* colony was initiated in 2000 from a single apterous parthenogenetic female from the clone Me LB05 (INRA-INSA Villeurbanne, France). Aphid rearing conditions were 20±1°C, 60±10% RH, and LD 16∶8 h. Four day old aphids (second instar larvae) were used for parasitization.

*Aphidius ervi* (Haliday) parasitoids (Hymenoptera: Aphidiidae) were purchased in 2010 from BIOBEST (Orange, France) as mummies. Upon reception, they were placed in plastic tubes (95×25 mm) plugged with cotton wool until emergence and were maintained in a climate chamber in the same conditions as their host *M. euphorbiae*, *i.e.* 20±1°C, 60±10% RH, and LD 16∶8 h, unless otherwise stated (heat stress). From emergence, adults were fed *ad libidum* with a 1∶1 honey/water solution until used for the experiments. In order to allow mating, groups of 30 newly emerged adult parasitoids (10 males and 20 females) were kept in tubes of 95×25 mm for two days, after which they were placed individually in smaller plastic tubes (75×12 mm).

### Heat Stress (HS)

Parasitoids emerging from the mummies (hereafter called G0 wasps) were submitted to a single heat stress consisting of transferring the young *A. ervi* adults from a climate chamber at the reference constant temperature of 20±1°C to incubators (SNIJDERS, Economic Premium ECP01E) at either 25±0.5°C or 28±0.5°C (HS25°C and HS28°C, respectively) for a period of 1 h or 48 h (HS_1h_ and HS_48h_, respectively). Therefore, four different heat stress treatments were applied: HS_1h_25°C, HS_1h_28°C, HS_48h_25°C and HS_48h_28°C. Controls consisted of parasitoids kept in the climate chamber at the reference temperature of 20°C. Prior to the heat stress treatments, all parasitoids were kept in the climate chamber at 20±1°C and fed *ad libidum*. Heat stresses of 1 h were performed on four-day old parasitoids and heat stresses of 48 h were performed on two-day old ones, so that all parasitoids were four days old when used for parasitization or measurements. This timing for heat stressing and testing parasitoids at the same age was deliberately chosen as the best compromise, even if the factor “heat treatment” could become mixed with the factor “parasitoid age”. G0 parasitoids surviving the heat stress, and those from control groups were then assessed for their longevity or used to parasitize aphids in order to investigate the G1 life history traits.

Climate chambers and incubators were regulated to be on the same photoperiod (LD 16∶8 h) and with similar relative humidity conditions (60±10%).

### Mortality Rate of *A. ervi* Wasps (G0) Induced by a Heat Stress and Longevity of the Surviving Individuals

Two days after their emergence, equal numbers of the mated male and female parasitoids were randomly assigned to one of the four HS treatment (HS_1h_25°C, HS_1h_28°C, HS_48h_25°C and HS_48h_28°C) or the control treatment (20°C), and HS treatments were applied as specified above. This random assignment was done nine or ten times, i.e. at different dates (9 for the 20°C treatment and ten for the 25°C and 28°C treatments), This allowed us to have the large sample sizes required to test differences in rates. Thus, globally between 118 and 168 individuals were tested in each HS and control treatment. At the completion of the HS or after 4 days at 20°C (controls), dead and surviving parasitoids were counted. The surviving parasitoids were kept individually in plastic tubes (75×12 mm) until death at 20°C, fed *ad libidum* (with a 1∶1 honey/water solution) and mortality was checked twice a day until all the animals were dead. Longevity of survivors was computed from the day of emergence until the day of death.

### Effects of a Heat Stress Applied to *A. ervi* Female Wasps (G0) on the Life-history Parameters of their Progeny (G1)

To obtain G1 wasps, aphids were parasitized by the surviving G0 wasps under controlled conditions. Preliminary experiments had been conducted in order to evaluate the potential effect of heat stresses on the probability of laying an egg in each attacked host (probability of true oviposition) under experimental conditions of controlled ovipositions. The procedure for controlled ovipositions consisted in placing a single four-day old, heat stressed or control *A. ervi* female, with 20 four-day old aphids on a potato leaf set on an agar layer in a 5 mm diameter Petri dish. When one aphid was stung by the parasitoid female, it was immediately removed and replaced by a new unparasitized one. For each HS treatment and the control treatment, ten A. *ervi* females were used, each being allowed to parasitize 9 to 14 aphids. The stung aphids had been dissected under a stereomicroscope immediately after oviposition and were checked for the presence or absence of a parasitoid egg. Never more than one parasitoid egg has been found in the stung aphids. The probability of true oviposition was systematically around 80% (HS_1h_25°C: 80.33%; HS_1h_28°C: 81.81%; HS_2J_25°C: 75.29%; HS_2J_28°C: 80.94%) and no significant effect of heat stress had been found (F_4,43_ = 0.99, *P* = 0.42). Therefore, this procedure of controlled oviposition could be applied to all HS treated females and control females.

To study the effects of a heat stress applied to *A. ervi* female wasps (G0) on the life-history parameters of their progeny (G1), controlled ovipositions by heat stressed or control *A. ervi* females were performed on successive dates. Depending on the numbers of emerging parasitoids, on a specific date, an equal number of females from the four HS treatments (HS_1h_25°C, HS_1h_28°C, HS_48h_25°C and HS_48h_28°C) or the control treatment (20°C) was used. For a total of 7 dates of experiments, a total of 190 females were used (N = unit of replication = 7, except for the 20°C reference treatment where N = 6). Each *A. ervi* female parasitized *ca.* 10 aphids. On a specific date, all aphids parasitized by females submitted to one of the five treatments were placed together in the same Petri dish (9 mm diameter) on a potato leaf set in agar and were maintained at the reference temperature of 20°C to complete their development. They were observed daily until emergence of the G1 parasitoid wasps. All dead aphids (*i.e.* aphids that had changed their usual colour and not moving under mechanical stimulation) were dissected under a stereomicroscope (Leica M165C) and the number of immature dead parasitoids was recorded. A larva was recorded as dead when found motionless following mechanical stimulation in the dissected body of a dead aphid.

The following parasitoids’ life-history parameters were recorded:

- Developmental time (from egg laying to adult emergence) in days.
- Total developmental mortality rate (%): ((No. of dead aphid nymphs containing a dead parasitoid larva+No. of dead mummies)/(No. of attacked aphids))×100.
- Mummification rate (%): (No. of formed mummies/No. of attacked aphids)×100.
- Emergence rate (%): (No. of mummies from which adult parasitoids emerged/No. of formed mummies)×100.
- Percentage of parasitoids completing total development: (No. of emerged parasitoids/No. of attacked aphids)×100.
- Sex ratio (%): (No. of males/No. of (females+males))×100.
- Egg load at emergence: Newly emerged females (less than 1 h) were anesthetized for one minute with ether then dissected into a drop of Phosphate Buffered Saline (PBS 1X) to collect their ovaries. The total number of mature eggs present in the two ovaries was recorded. The length (µm) of G1 females’ thorax was measured using a stereomicroscope.

In addition, the percentage of hosts surviving after G0 female parasitoid attack ((No. of aphids that did not form mummies/No. of attacked aphids)×100) was also computed.

### Statistical Analysis

#### Effect of HS on G0 life history traits

To test the effects of duration of stress (1 h or 48 h), temperature of stress (20°C, 25°C or 28°C) and sex on mortality rate of *A. ervi* wasps (G0) submitted to a heat stress, we used a GLM with binomial error (logit link) as a binomial distribution is appropriate to model binary data or percentages. The three explanatory variables and their interactions were included in the full model.

Longevity of G0 individuals that survived the heat stress was analyzed by the means of a GLM (Gamma error, inverse link). Gamma is a continuous probability distribution that is frequently used to model waiting time since it has positive value and its two parameters (shape and scale) allow it to fit with large data distributions [40]. Duration of stress, temperature of stress and sex were included with all the interactions in the statistical model as explanatory variables (qualitative variables).

#### Effects of HS applied to G0 female wasps on their progeny traits (G1 wasps)

To test for the effects of temperature and duration of stress and sex on G1 parasitoid development time, the generalised estimating equation (GEE) method was used, assuming a Gamma distribution of errors and an exchangeable working correlation structure (other working correlation structures yielded similar results). The GEE method accounts in generalized linear models for the correlation between observations [41]. Indeed, on a specific date, all aphids parasitized by females submitted to a given HS treatment were placed together until the end of their development (see section “Effects of a heat stress applied to *A. ervi* female wasps (G0) on the life-history parameters of their progeny (G1)”). This group effect was included as a random factor in the GEE, the other explanatory variables were considered as fixed effects. Likelihood-based methods are not available for estimating levels of significance in GEE methods. The Wald statistic, based on the asymptotic normality of estimators, was used instead.

Effects of temperature and duration of stress and thorax size on egg load at emergence of G1 females were also analyzed by GEE models, but assuming a Poisson distribution of errors (log link). The log link ensures that all the fitted values are positive while the Poisson distribution takes into account the fact that the data are integers. Thorax size was added as the first explanatory variable in the model to remove any potential effect its correlation with egg load could have.

We used a generalised linear model with a binomial error (logit link) to analyze the effects of temperature and duration of stress on i) the percentage of G1 parasitoids completing development (No. of emerged parasitoids/No. of attacked aphids), ii) the developmental mortality rate (No. of dead aphid nymphs containing a dead parasitoid larva+No. of dead mummies)/(No. of attacked aphids), iii) the mummification rate (No. of formed mummies/No. of attacked aphids), iv) the emergence rate (No. of mummies from which adult parasitoids emerged/No. of formed mummies), v) the sex ratio, defined as the percentage of males in the progeny, vi) the percentage of hosts surviving after parasitization (No. of aphids that did not form mummies/No. of attacked aphids).

For all the statistical models we performed, we fitted all the main effects and the interaction terms for each data set. We followed a backwards procedure to remove variables sequentially, thus allowing us to identify the most parsimonious model. Data were analyzed using the statistical software package R [42]. The R package ‘*geepack*’ and the function *geeglm* were used for GEE analyses.

## Results

### Mortality Rate of *A. ervi* Wasps (G0) Induced by a Heat Stress and Longevity of the Surviving Individuals

The percentage of G0 wasps that died before the completion of the HS was significantly affected by the temperature in interaction with the duration of the stress (Chi^2^ = 136.01, df = 2, *P* = 0.001). An increase of HS temperature increased the mortality when the stress was applied for 48 h (from 18.13± (SEM) 4.78% at 20°C to 47.48±9.46% at 25°C and to 59.00±9.73% at 28°C) while there was little or no effect of the temperature when the stress was applied for 1 h (21.84±4.94% at 25°C and 18.18±5.32% at 28°C). The mortality of G0 wasps was not sex dependent. The triple and other double interactions were not significant.

**Figure 1:**
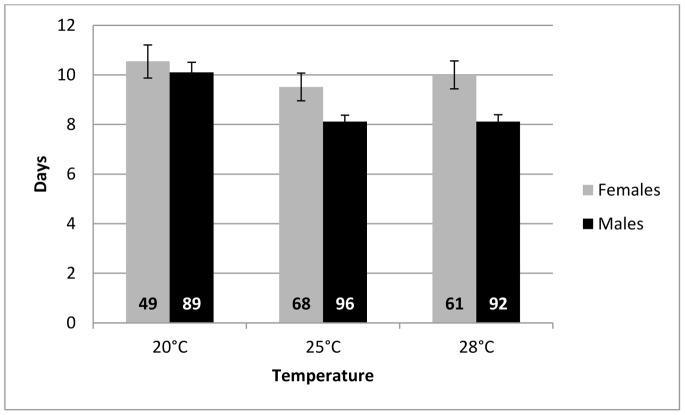
Mean longevity (days) of G0 *A. ervi* individuals surviving the heat stress. This figure represents the mean longevity (± SEM) of G0 *A. ervi* males and females that survived the heat stress (25°C or 28°C), compared to individuals placed in the 20°C control treatment. Since the duration of the stress (1 h or 48 h) had no significant effect on longevity, data were pooled (cf. text for details). All parasitoids were fed *ad libitum* until death. The number of tested males (black bars) and females (grey bars) is written at the base of each bar.

Longevity of surviving G0 individuals was negatively affected by the temperature of the heat stress (Chi^2^ = 96.53, df = 2, *P*<0.001) and surviving females lived longer than surviving males (Chi^2^ = 94.69, df = 1, *P*<0.001, Fig. 1). There was no statistical difference between male and female mean longevity for the control treatment. Duration of stress was not significant nor were all the possible interactions between explanatory variables. In details, mean longevities (in days (SEM)) were, for males: 10.10 (0.41) for control, 8.28 (0.35) for HS_1h_25°C, 8.40 (0.36) for HS_1h_28°C, 7.88 (0.41) for HS_48h_25°C and 7.50 (0.48) for HS_48h_28°C; for females: 10.54 (0.67) for control, 9.73 (0.78) for HS_1h_25°C, 10.18 (0.69) for HS_1h_28°C, 9.10 (0.68) for HS_48h_25°C and 9.53 (0.93) for HS_48h_28°C.

### Effects of a Heat Stress Applied to *A. ervi* Female Wasps (G0) on the Life-history Parameters of their Progeny (G1)

#### G1 Developmental time

**Figure 2:**
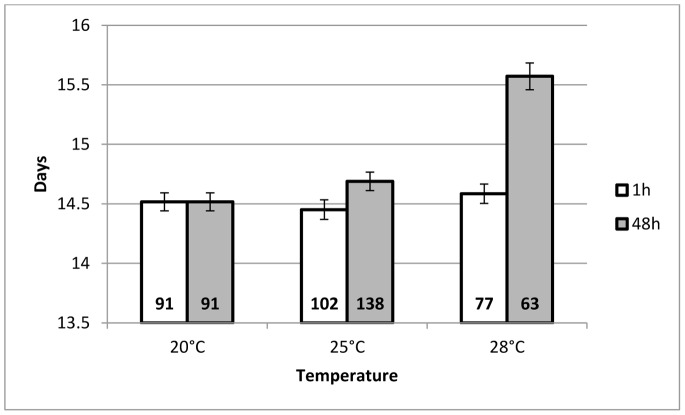
Mean developmental time (days) of G1 *A. ervi* progeny. This figure represents the mean developmental time (± SEM) of G1 *A. ervi* wasps whose mothers had been submitted to a heat stress, for a duration of either 1 h (white bars) or 48 h (grey bars), at a temperature of 25°C or 28°C, compared to progeny of the females that underwent the 20°C control treatment. Since the factor “sex” had no significant effect, data from males and females were pooled (cf. text for details). The number of individuals is written at the base of each bar.

Progeny developmental time was significantly different between males (mean = 14.55 days, SEM = 0.04) and females (mean = 15.17 days, SEM = 0.08) (Chi^2^ = 53.8, df = 1, *P*<0.001) and was affected by the temperature in interaction with duration of the HS (Chi^2^ = 22.8, df = 2, *P*<0.001). An increase in the duration from 1 h to 48 h induced a significantly longer development when mothers were heat stressed at 28°C compared to 25°C or to 20°C (Fig. 2).

Mean developmental times (in days (SEM)) were, for males: 14.40 (0.08) for control, 15.40 (0.08) for HS_1h_25°C, 14.5 (0.10) for HS_1h_28°C, 14.60 (0.08) for HS_48h_25°C and 15.33 (0.24) for HS_48h_28°C; for females: 15.00 (0.15) for control, 15.78 (0.26) for HS_1h_25°C, 14.93 (0.19) for HS_1h_28°C, 15.03 (0.17) for HS_48h_25°C and 16.04 (0.07) for HS_48h_28°C.

#### G1 Total developmental mortality rate

There was a significant effect of the temperature of the stress on developmental mortality rate, with mortality increasing when temperature increased (Chi^2^ = 9.58, df = 2, *P* = 0.008): the mean developmental mortality rate was 48.51% (95% CI = 7.90) at 20°C, 51.51% (95% CI = 7.29) at 25°C and 53.38% (95% CI = 5.66) at 28°C. There was no significant interaction between temperature and duration, nor an effect of duration.

#### G1 Mummification rate

**Figure 3:**
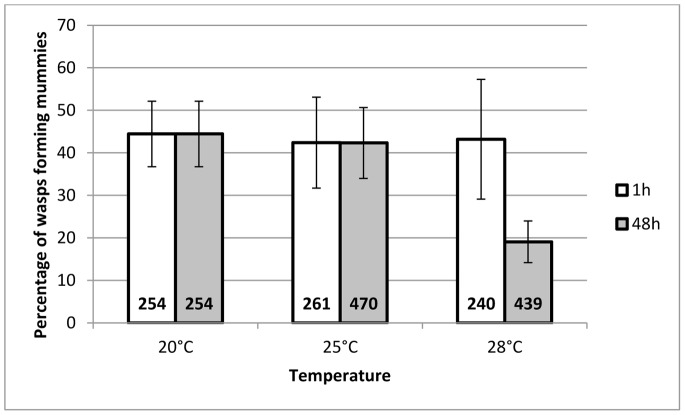
Mean mummification rate (%) of G1 *A. ervi* wasps. This figure represents the mean percentage (±95% CI) of G1 *A. ervi* wasps forming mummies, whose mothers had been submitted to a heat stress for a duration of either 1 h (white bars) or 48 h (grey bars), at a temperature of 25°C or 28°C (N = 7), compared to progeny of the females that underwent the 20°C control treatment (N = 6). The total number of attacked aphids monitored is written at the base of each bar.

There was a greater decrease in the rate of progeny mummification for a HS duration of 48 h than for a HS duration of 1 h when the temperature increased (interaction between temperature and duration of the HS: Chi^2^ = 22.8, df = 2, *P*<0.0001; Fig. 3).

#### G1 Emergence rate

**Figure 4:**
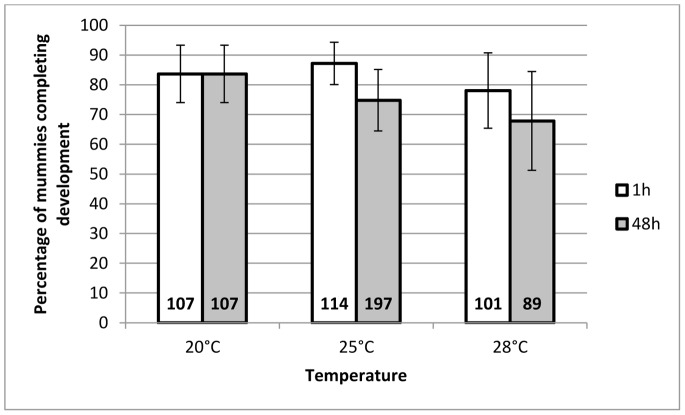
Mean emergence rate (%) of G1 *A. ervi* mummies. This figure represents the mean percentage (±95% CI) of G1 *A. ervi* mummies completing nymphal development, whose mothers had been submitted to a heat stress for a duration of either 1 h (white bars) or 48 h (grey bars), at a temperature of 25°C or 28°C (N = 7), compared to progeny of the females that underwent the 20°C control treatment (N = 6). The total number of monitored mummies is written at the base of each bar.

Emergence rate significantly decreased with the duration of the HS in interaction with the temperature of the HS, (Chi^2^ = 22.9, df = 2, *P*<0.0001). For a HS duration of 1 h, emergence rate decreased only at 28°C while for a HS duration of 48 h, the decrease was observed at 25°C (Fig. 4).

#### Percentage of G1 parasitoids completing total development

**Figure 5:**
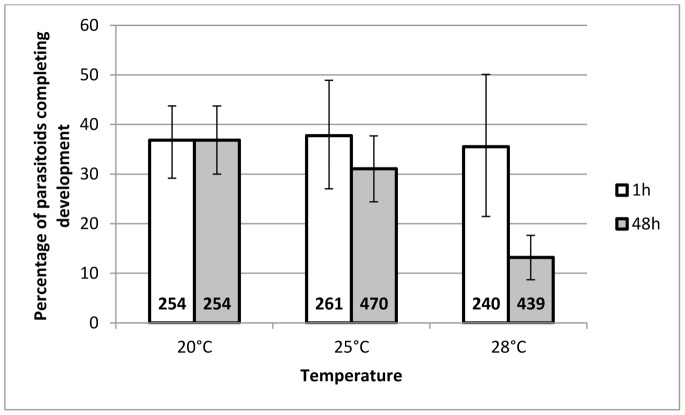
Mean percentage of G1 *A. ervi* completing total development. This figure represents the mean percentage (±95% CI) of G1 parasitoids completing total development, whose mothers had been submitted to a heat stress for a duration of either 1 h (white bars) or 48 h (grey bars), at a temperature of 25°C or 28°C (N = 7), compared to progeny of the females that underwent the 20°C control treatment (N = 6). The total number of attacked aphids monitored is written at the base of each bar.

The percentage of parasitoids completing total development was significantly affected by the interaction between temperature and duration of the HS (F_2,34_ = 3.41, *P = *0.045) (Fig. 5). A 1 h HS had little or no effect whereas a 48 h HS induced a strong decrease in the percentage of parasitoids completing development.

#### Sex ratio (% of adult males) of G1 parasitoids

There was no modification of the sex ratio due to the temperature or duration of the HS (mean % ±95% CI) (20°C: 80.40±9.70, HS_1h_25°C: 88.30±7.86, HS_48h_25°C: 80.81±8.80, HS_1h_28°C: 79.58±21.54 and HS_48h_28°C: 71.34±21.03). The interaction between the two factors was not significant.

#### Egg load at emergence for G1 wasps

Thorax size had a significant effect, with larger females producing a larger number of eggs at emergence (Chi^2^ = 13.1, df = 1, *P*<0.001). After removing the effect of thorax (see data analysis), temperature of the HS also had a significant effect (Chi^2^ = 45.9, df = 2, *P*<0.01). The mean number of mature eggs in the ovaries of newly emerged G1 females was significantly greater when their mother had been submitted to a HS of either 25°C (mean = 112.25, SEM = 6.04) or 28°C (mean = 105.35, SEM = 7.39), compared to the reference treatment of 20°C (mean = 90.00, SEM = 4.43). Interactions and other additive effects were not significant.

#### Percentage of hosts surviving after G0 female parasitoid attack

**Figure 6:**
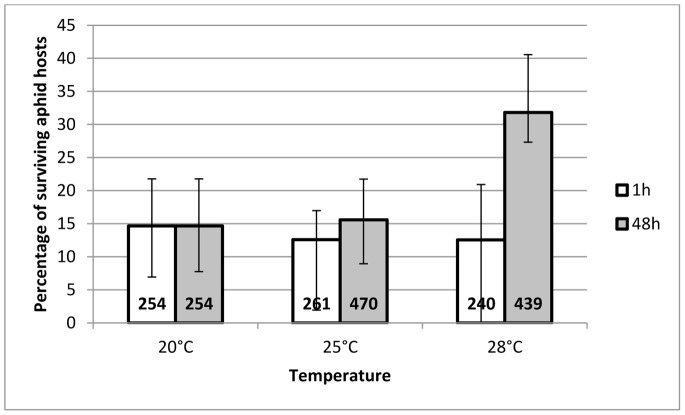
Mean percentage of aphid hosts surviving after G0 *A. ervi* **females attack.** This figure represents the mean percentage (±95% CI) of aphid hosts surviving after an attack by G0 *A. ervi* females that had been submitted to a heat stress for a duration of either 1 h (white bars) or 48 h (grey bars), at a temperature of 25°C or 28°C (N = 7), compared to hosts attacked by G0 *A. ervi* females that underwent the 20°C control treatment (N = 6). The total number of attacked aphids monitored is written at the base of each bar.

There was a significant interaction between duration and temperature of the HS (Chi^2^ = 10.78, df = 2, *P* = 0.004). For a HS duration of 1 h, the percentage of hosts surviving after G0 parasitoid attack did not change. However for a HS duration of 48 h, there was a clear increase in this percentage at 28°C (Fig. 6).

## Discussion

### Heat Stress Affects Adult Wasp Survival

The heat stresses performed for 48 h had the greatest impacts on the wasps’ mortality, inducing *ca*. 30% (HS25°C) to 40% (HS28°C) additional mortality when compared to controls. The lifespan of the surviving G0 *A. ervi* was reduced when the temperature increased, with males being more affected. Insect metabolic rate depends on temperature which influences their longevity so that insects live for a shorter time at higher temperatures [10], [43]. Therefore, the negative effect of a heat stress on *A. ervi* male wasps’ longevity is likely to be due to immediate and direct effects of temperature on the insect physiology. Also, the heat stress procedure may have resulted in a selection of the fittest individuals, with males and females responding differently. Study of another *Aphidius* species also showed that the longevity after a 1 h heat shock at 36°C was sex-dependant [22]. Fewer males survived than females after the heat shock and their longevity decreased compared to controls. Conversely, heat shocked females lived longer than controls, possibly due to the fact that only the most resistant individuals survived the stress. It is suggested that females may be more resistant to a heat stress because of their larger size, together with their diploid status conferring a better chance to respond to changes [22]. It is worthy to note that parasitoid males are often studied less than females and their role underestimated. The reduction in their longevity, induced by a heat stress, may not have consequences on their fitness, as they usually mate very quickly after emergence. However, it would be interesting to evaluate their fecundity, both in terms of quantity and quality of spermatozoids.

Finally, G0 immediate mortality following a heat stress was not sex dependent, in contrast to the longer term effects on longevity that were more drastic in males than in females. Therefore, the present study also highlights the need to consider both immediate and longer term effects following a stress.

### Exposing Female Wasps to Heat Stress Negatively Impacts Certain Traits of their Progeny

The major result emerging from this study is that a heat stress applied to *A. ervi* parents did not only affect them but also their progeny whose developmental time, rates of mummification, rates of emergence and percentage of parasitoids completing total development were negatively affected. Also, some life history traits varied depending on the heat stress their mother was subjected to, although all developing parasitoids were kept at the constant temperature of 20°C.

The percentage of parasitoids completing total development was significantly reduced by more than a half when *A. ervi* mothers were submitted to the strongest heat stress, *i.e.* 48 hours at 28°C. The success of the parasitoid development depends on the success of both stages of mummification and emergence. Since the rate of adult wasps emerging from mummies was significantly but only slightly affected by the HS (with a maximum of a 16% decrease for the HS_48h_28°C), the strong decrease in the overall success of parasitoid development must be mainly attributed to the mortality of the host-parasitoid couple occurring before mummification. This suggests that both embryonic and larval instars of *A. ervi,* whose viability is negatively affected when their mother wasp is submitted to a HS, represent the developmental stages the most sensitive to environmental variations. In another work where *A. ervi* was exposed to sublethal conditions of abiotic stress (insecticide), the pre-nymphal stages also turned to be the most sensitive ones [44]. Similarly, the aphid parasitoid *Binodoxys communis* was also mainly affected at these precocious developmental stages after the aphid hosts had ingested toxic plant secondary metabolites [45]. In the present study, the decrease in successful completion of development in *A. ervi* whose mothers had been exposed to a HS could also be partly explained by a higher survival of their aphid hosts. All heat stress treatments were applied to the female parasitoids before but also during their oviposition in aphids, so that aphids were also shortly heat stressed at the same temperature for a maximum duration of 10 minutes. Effects of temperature on aphid resistance to parasitoids mediated by secondary symbionts have been demonstrated [24]. However, no known symbionts were found in our clone of *M. euphorbiae* (data not shown). Also, as the probability of true oviposition by *A. ervi* mothers in an attacked host did not differ between treatments, the proportion of parasitized aphids at the beginning of each experiment could be considered as equal. As a result, any difference in percentages of aphids evading parasitism might not be due to cases of unparasitized hosts, but rather to an increased percentage of parasitoid eggs or larvae failing to complete their development. However, as this increased survival of attacked hosts was only observed for a HS of 48 h at 28°C, a possible physiological suppression of the developing parasitoids by the aphid hosts seems very improbable.

Instead, the HS is more likely to affect the female wasps’ physiology, at least their reproductive system. Indeed, a heat stress on the parasitoid mother before parasitization may affect its virulence qualitatively and/or quantitatively, and subsequently favour the resistance of the host. A heat stress may alter virulence factors of *A. ervi* such as female wasps’ venom and ovarian secretions which are responsible for the alteration of the host physiology in favour of the parasitoid development [46]. The heat stress may also affect the viability of the eggs and/or their ability to release teratocytes, thus decreasing their chance of successful development [47]. Such alterations could be related to the significant increased duration of development observed following a HS_48h_ 28°C on *A. ervi* mothers. More studies are needed in order to better understand how host/parasitoid interactions are affected when the parasitizing mother wasp has been heat stressed prior to and during oviposition.

### Exposing Female Wasps to Heat Stress Positively Impacts Female Progeny Egg Load at Emergence

Surprisingly, whereas most G1 development related traits were negatively affected, the total number of mature eggs at emergence of the female progeny increased when their mother had been submitted to a heat stress of 25°C or 28°C. We propose this could result from homeostatic modulation implying some biological plasticity of the developing parasitoids. Indeed, environmental changes are often reported to induce trade-offs in the allocation of resources among different physiological processes. Increased allocation to certain processes - *e.g.* growth - is expected to result in decreased allocation to other ones - *e.g*., metabolism and/or reproduction – or inversely [48], leading to either positive or negative consequences for fitness. In this regard, our results suggest that trade-offs might have occurred between developmental traits and adult female egg production at emergence. A heat stress applied on the mothers could have allowed such trade-offs to be fulfilled throughout the whole developmental window of its progeny, which may then have resulted in an improved fitness of the emerging daughters. The individuals whose mothers had received a 48 h heat stress took longer to complete development, which, in female progeny, could have benefitted egg maturation, therefore resulting in high egg loads at emergence. Another likely explanation could be that only the most robust progeny from the heat stressed mothers survived throughout the whole development period, resulting in the selection of females also having a greater egg load at emergence. Altogether, heat-stressing female wasps resulted in the production of daughters that were either more pro-ovigenic or that presented a greater potential for a higher future realized fecundity. Monitoring female progeny during their whole life could provide interesting information regarding this point.

### *A. ervi* Wasps Exhibit Trans-generational Phenotypic Plasticity: Implications for Biological Control

Abiotic stresses such as temperature changes are common, and adaptations to stress are expected to occur in natural populations. In the fields or in greenhouses, insects are frequently exposed to mild/hot variations of the temperature in the range of the ones used in this study (25°C–28°C) and for similar lengths of time (1 h–48 h). The environment that a mother experiences often affects offspring phenotype through trans-generational phenotypic plasticity [4]. Here, both parental and trans-generational effects were observed, thus revealing the complexity of insect responses and underlying mechanisms to fluctuating conditions of their natural environment. Trans-generational phenotypic changes are believed to be adaptive as organisms with the induced phenotype experience a greater fitness [49]. In our study, the greater egg load at emergence observed in the wasp progeny, following a parental heat stress, could contribute to improve their overall fitness. Conversely, the greater egg load at emergence could also be detrimental to other fitness traits such as longevity, ultimately resulting in an unchanged or even decreased total egg load (realised fecundity) compared to the progeny of non-heat-stressed wasps. To test these hypotheses, it would be interesting to observe the egg laying behaviour and to measure the realised fecundity and longevity of the progeny [50].

Maternal effects may also have occurred. These maternal effects are the product of cross-generational interactions between parental phenotype, parental environment and offspring genotype and are expressed as phenotypic differences in the offspring [51]. Further studies will compare the proteomics of mothers ‘secretions and eggs produced under each heat stress condition. Analysis of protein expression following such mild heat stress should help in unravelling the physiological mechanisms underlying the trade-offs possibly occurring during progeny development.

Finally, these findings are of importance when assessing the efficiency of parasitoid insects as biological control agents. For this study, we used aphids from a single clone as hosts for *A. ervi* parasitoids, in order to overcome any variability associated with the host and better take into account the potential variability exhibited by the parasitoid. We also deliberately chose to work on a strain of *A. ervi* that is being reared and commercialized for agricultural and horticultural purposes, even if the possibility that the individuals from the strain used in this study may respond differently from wild ones cannot be ruled out. For instance, a parental stress of HS_48h_ 28°C applied to *A. ervi* females was shown to induce an increase of *ca.* one day of the progeny total developmental time, which could in turn directly affect the populations’ dynamics.Our study underlines the fact that even small variations of the environment can influence the quality and efficiency of parasitoid insects. Indeed, other studies emphasise the role of thermal factors in host-parasite interactions. They mostly report that individuals subjected to hot or cold thermic stresses are directly negatively impacted, particularly regarding key physiological parameters associated with their fitness [13]–[17], [19]–[23]. The present work highlights the fact that, not only potential beneficial effects could occur but, above all, that some effects can be expressed at the next generation. Therefore, the effects of thermal stresses on parasitoids should be more extensively considered and their study should integrate trans-generational investigations. Taking into account such trans-generational phenotypic plasticity could help improving the application of biocontrol programmes.
